# Contrast-enhanced 3D micro-CT of plant tissues using different impregnation techniques

**DOI:** 10.1186/s13007-017-0256-5

**Published:** 2017-11-28

**Authors:** Zi Wang, Pieter Verboven, Bart Nicolai

**Affiliations:** 1 Division MeBioS, Department of Biosystems, KU Leuven – University of Leuven, Willem de Croylaan 42, 3001 Leuven, Belgium; 2 Flanders Centre of Postharvest Technology, Willem de Croylaan 42, 3001 Leuven, Belgium

**Keywords:** Contrast agent, Porosity, Microstructure, Image analysis, Cesium iodide, Vasculature, Cell size, Tomato, Apple, Pear

## Abstract

**Background:**

X-ray micro-CT has increasingly been used for 3D imaging of plant structures. At the micrometer resolution however, limitations in X-ray contrast often lead to datasets with poor qualitative and quantitative measures, especially within dense cell clusters of plant tissue specimens. The current study developed protocols for delivering a cesium based contrast enhancing solution to varying plant tissue specimens for the purpose of improving 3D tissue structure characterization within plant specimens, accompanied by new image processing workflows to extract the additional data generated by the contrast enhanced scans.

**Results:**

Following passive delivery of a 10% cesium iodide contrast solution, significant increases of 85.4 and 38.0% in analyzable cell volumes were observed in pear fruit hypanthium and tomato fruit outer mesocarp samples. A significant increase of 139.6% in the number of analyzable cells was observed in the pear fruit samples along the added ability to locate and isolate better brachysclereids and vasculature in the sample volume. Furthermore, contrast enhancement resulted in significant improvement in the definition of collenchyma and parenchyma in the petiolule of tomato leaflets, from which both qualitative and quantitative data can be extracted with respect to cell measures. However, contrast enhancement was not achieved in leaf vasculature and mesophyll tissue due to fundamental limitations. Active contrast delivery to apple fruit hypanthium samples did yield a small but insignificant increase in analyzable volume and cells, but data on vasculature can now be extracted better in correspondence to the pear hypanthium samples. Contrast delivery thus improved visualization and analysis the most in dense tissue types.

**Conclusions:**

The cesium based contrast enhancing protocols and workflows can be utilized to obtain detailed 3D data on the internal microstructure of plant samples, and can be adapted to additional samples of interest with minimal effort. The resulting datasets can therefore be utilized for more accurate downstream studies that requires 3D data.

## Background

Composition and internal tissue structure dictates the physical and functional characteristics of plants [1]. Thus, anatomical parameters such as cell size, geometry, tissue composition and architecture must be known to understand the physiology of plants in detail [2–5]. Accurate three-dimensional (3D) anatomical data is necessary for modelling and understanding transport of water, nutrient and gases in plants. Thus, raw images in which the models are based on should be of high resolution, accuracy and throughput [6].

X-ray micro computed tomography (X-ray micro-CT) has become popular for 3D plant tissue imaging [7–9]. Generational improvements to CT hardware and software has made the technique popular [10] with the scientific community since its initial introduction to medical imaging in 1972 [11]. Given that benchtop instruments have resolutions down to half a micrometer [12], contrast in water saturated specimens becomes a limiting factor in the determination of internal 3D microstructure instead of resolution. Thus, the ability to determine structural boundaries within dense clusters becomes exceptionally difficult [13]. Only with phase contrast imaging from synchrotron radiation, has micro-CT been able to somewhat overcome this problem, albeit at extreme expense [14, 15].

In principle, contrast of X-ray images is dependent on density, thickness and atomic composition of the sample [16]. Since biological samples are typically composed of low atomic weight elements, differential introduction of heavy elements into the sample can exponentially increase the attenuation of X-ray beams through the structures of interest. More specifically, the attenuation of the X-ray beam is approximately proportional to the cubic of the average atomic number [17]. Medical imaging currently exploits this concept through the use of contrast agents which have heavy elements embedded within [18].

Due to toxicity concerns, clinical contrast agents are based on barium sulfate or iodide compounds [19]. Which were also formulated with osmolality, iconicity and viscosity matching the requirements of human patients. As such, the use of medical contrast solution for plant specimens are limited as they were never designed to do so. Moreover, medical contrast agents are categorized as medical supplies, and are therefore restricted to radiology specialists. This further reduces its feasibility for use within the plant science domain.

To counter this limitation, a number of iodine and heavy metal compounds were explored for their viability for use within plant specimens [20]. However, results highlighted some significant shortcomings of these compounds along with their methodologies. For example, Lugol’s solution causes significant tissue damage in the incubated samples, whereas phosphotungstic acid is prone to leeching from the sample and is toxic [21]. Bismuth tartrate requires extended incubation time and osmium tetroxide has poor penetrative properties while being extremely expensive and toxic [21]. Moreover, previously adapted protocols require extensive sample preparation including dehydration and fixation along with long incubation time with iodine contrast [22]. This automatically excludes plant specimens with high water content as viable samples.

So, the most ideal contrast agent for widespread usage should be readily accessible, non-toxic, inexpensive along with a simple and short incubation protocol. In theory, a solution with a heavy cation would suffice considering that the middle lamella that is present at the intercellular interface is rich in natively charged pectin [23]. Working from the heaviest to lightest metallic elements on the periodic table that have ionic compounds with these aforementioned properties. Cesium salts thus remain as one of the few metallic compounds that can potentially be used as a contrast enhancing agents. Considering that cesium salt variants comes with chloride, iodide and fluoride anions, care must be taken to choose the most compatible compound. However, the chloride and fluoride variants have relatively light anions, and they have the potential to be dehydrating and cytotoxic respectively. Therefore, cesium iodide emerges as a prime candidate as it in theory meets all the aforementioned requirements and both its cation and anion are composed of heavy elements. Moreover, it is reasonable to theorize that cesium cations will gravitate toward the charged pectin molecules providing contrast enhancement to cell boundaries. While the anion embedded solution should in theory diffuse through intercellular water and enhancing its contrast. However, the necessary concentration, incubation method and duration for successful utilization of the theorized contrast agent is unknown.

Thus, the potential benefits of utilizing cesium iodide as a contrast enhancing agent for X-ray micro-CT scans of plant tissues were experimentally tested. The contrast delivery protocols were optimized for use with commercially relevant fruit species, with an emphasis on minimal tissue damage to preserve scan accuracy. Additionally, plant specimens of varying intercellular air fractions were examined for use with the cesium iodide to determine usage feasibility as well as necessary specimen specific adjustments.

## Methods

### Sample preparation and contrast incubation

Hypanthium samples of “Kanzi” apples (*Malus domestica* cv. Kanzi) and “Conference” pears (*Pyrus communis* cv. Conference) harvested during the fall of 2015 were obtained from internal stock and purchased fresh from a local market respectively. The fruits were stored at 4 °C until used for experimentation (less than 7 days post acquisition). Hypanthium samples were extracted from the fruit via a cork borer with an inner diameter of 4.05 mm, and the top 8 mm of the core sample was kept for experimentation and imaging.

Outer mesocarp samples were excised from greenhouse “Bonaparte” tomatoes (*Solanum lycopersicum* cv. Bonaparte) while petiolule and leaflet blade sections were obtained from the “Merlice” variety. Greenhouse tomatoes were picked prior to ripening at the first sign of color change, stored at room temperature and were used for experimentation within 1 day of picking. Mesocarp samples were excised by hand via a razor blade and samples approximately 4 × 4 × 7 mm were used for experimentation and imaging. Leaflet blade sections were obtained from leaves approximately 50 mm at the widest point in 5 × 8 mm sheets with 300 µm maximal thickness. Petiolule samples were cut in 10 mm long segments up to 50 mm from the leaflet blade, and imaged sections were approximately 2.5 mm in diameter.

In all experiments, plant samples were scanned with or without contrast treatment along with protective parafilm to prevent dehydration during imaging. All cesium iodide solutions (Acros Organics, Geel, Belgium) were prepared fresh prior to scan sessions. Final necessary concentration was experimentally derived using apple hypanthium samples.

Contrast delivery in fruit samples was done either passively via diffusion or actively via vacuum impregnation. The passive method applies the contrast solution by sample submersion at room temperature where the time frame for diffusive incubation was determined experimentally. If the sample can be successfully enhanced via the passive method, no additional methods were tested. In the event the passive method was insufficient, the active method was applied. In essence, the active method applies an extra pulsed vacuum profile similar to that utilized to impregnate leaf samples with trehalose [24, 25]. Principally, the experimentally derived pulsed vacuum profile replaces intercellular air with the contrast solution, thus making it easier for the contrast solution to fully diffuse throughout the sample.

Conversely, intact leaflet blade and petiolule samples were partially submerged to utilize the natural transpirational pull found in leaves and vasculature. Incubation time was optimized per sample type and experimentally determined with ambient conditions set at 22 °C and 30% RH. Leaflet and petiolule sections were excised for scanning following contrast incubation.

### X-ray micro-CT acquisition and reconstruction

All scans were acquired via a Phoenix Nanotom micro-CT system (General Electric, Heidelberg, Germany). Projection images were captured per sample on a 12-bit 2304 × 2304 detector with voxel resolutions of 2.5–3.0 µm (sample type dependent). X-ray tube voltages of 45–75 kV (sample dependent) were applied to capture 2400 projection images with an exposure time of 500 ms per projection, resulting in a 20 min scan time per sample. Octopus Reconstruction 8.9.2 (Inside Matters, Gent, Belgium) was used to for reconstruction utilizing a filtered back projection algorithm. Ring artifact and noise filters were applied to improve overall image quality. Reconstructed images were downscaled to 8 bits to reduce computational requirements during image processing.

### Image processing, segmentation and analysis

Processing workflow was derived from previously published works [5]. However, as contrast enhanced scans yield substantially better definition, the workflow was modified to better harvest the additional data. Sub-volumes were extracted from the reconstructed 3D volume for analysis, and all sub-volumes utilized for analysis were greater than the minimum representative volume of 1.3 mm^3^ as previously determined in apple hypanthium samples [26]. Due to the inevitable damage to the outer edges of the scanned samples, volume of interests (VOIs) of 2400 × 2400 × 2400 µm were utilized to analyze hypanthium samples. As tomato samples were even more delicate and damage prone, a reduced VOI of 2000 × 2000 × 2000 µm was utilized for analysis. However, as leaflet and petiolule samples are highly two dimensional and anisotropic, it was not possible to define a 3D VOI. Thus, representative images from undamaged regions were used to demonstrate the effect of the contrast solution.

Image segmentation was necessary to convert greyscale data to binary to reduce computational load and was done via a histogram based multi-thresholding module in Avizo 9.2 (FEI, Bordeaux, France) with assistance from the Sobel operator (edge detection to determine the approximate cell boundaries). Despeckling and opening operations were performed to reduce noise in the binary images, and segmented cells in fruit tissue were subjected to watershed transform for further object separation. Incomplete objects in contact with the VOI border were removed and a debris filter based on equivalent cell diameter was instituted to remove nonsensical objects. Debris filters of 40 and 80 µm were set for apple hypanthium and tomato mesocarp samples. However, due to the presence of brachysclereids [27], no lower filter was set for pear hypanthium samples. Regardless, all remaining objects were labelled and subjected to a more detailed 3D analysis for parameters, such as equivalent spherical diameter, sphericity, anisotropy, number of cells, analyzable cell volume (%). The effective cell diameter, 3D shape, directionality, quantity and gains in analyzable data were subsequently assessed and compared to control samples. In all fruit samples, 4 VOIs were analyzed to yield quantitative data.

Conversely, as the petiolule is a heterogeneous sample, image segmentation was adjusted to target visible cell structures. The workflow was similar to that of the fruit samples with the exception of the borderkill command. Moreover, the cuticle layer and the epidermis were removed to better visualize the internal structure of the petiolule.

### Errors induced by contrast solution incubation

To assess whether the contrast enhancement protocol altered the samples in any way, a pair of Kanzi apple scans were performed prior to and following contrast enhancement. The datasets were registered and volumetric analysis was done using Avizo. Pre-alignment of principal axes was performed to reduce compute time. Transformation parameters were set to rigid and aniso-scale, and the correlation metric was utilized for the image registration. A matching sub-volume of 1950 × 1950 × 1950 µm was extracted from both datasets to determine changes to the sample prior to and after contrast enhancement. An exclusive or (XOR) function was utilized to highlight the difference between the images and highlighted voxels were subjected to quantitative analysis.

### Statistical analysis

All quantitative comparisons between control and contrast treated samples were subjected to independent t tests with a sample size of four per analysis group. Statistical analysis was performed utilizing Prism 6 (GraphPad Software, La Jolla, USA). For normalized figures, statistical analysis was performed on the raw data. In all instances, statistical significance was stated if the p value was less than 0.05.

## Results

**Fig. 1:**
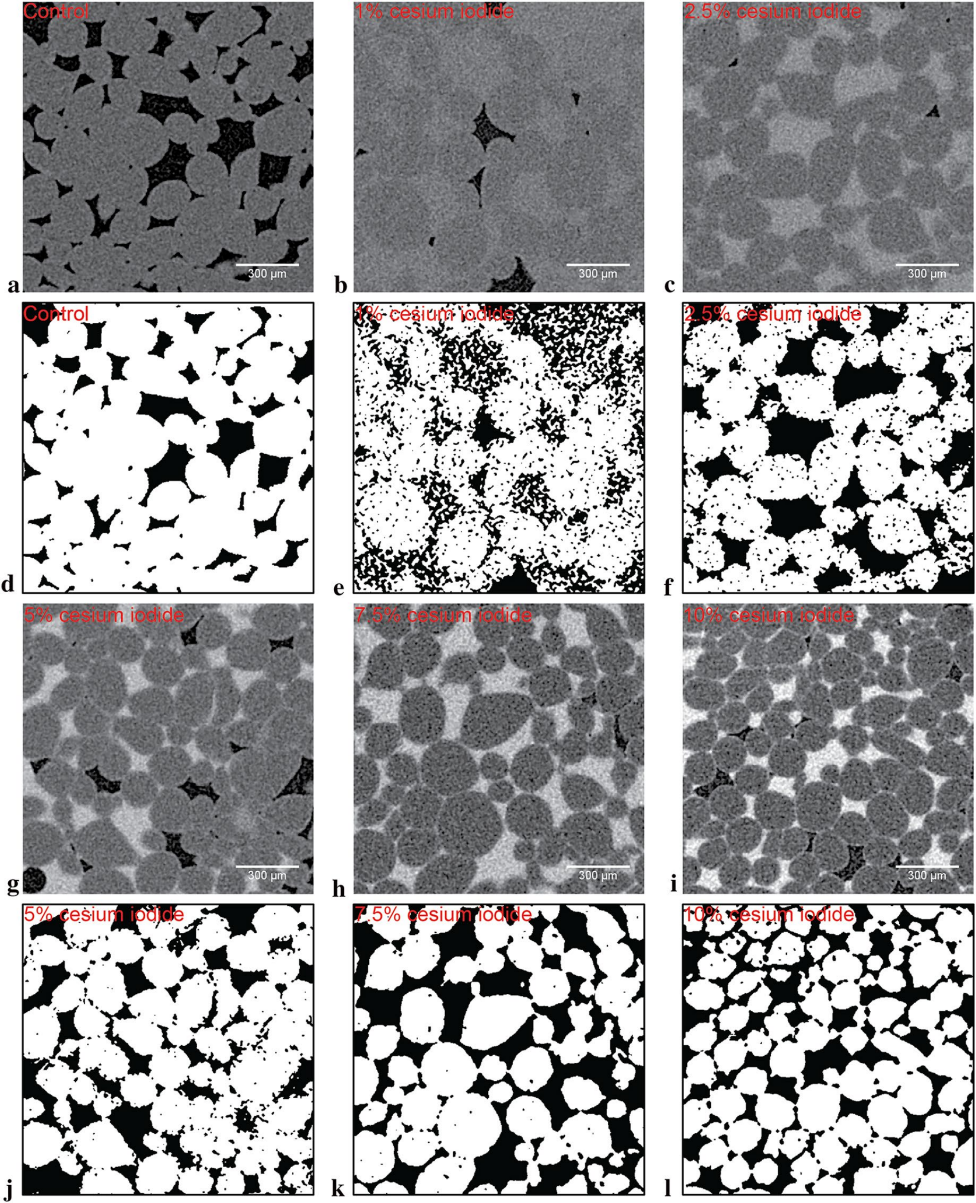
Contrast altering effects of varying concentrations of cesium iodide delivered to apple (cv. Kanzi) hypanthium. Greyscales (**a**–**c**, **g**–**i**) and segmented binary images (**d**–**f**, **j**–**l**) are sequentially of control, 1, 2.5, 5, 7.5 and 10% w/v cesium iodide embedded samples. In the control greyscale (**a**), intercellular air space and cells are designated by the black and dark grey pixels, respectively. Contrast embedded samples (**b**, **c**, **g**–**i**) have dark grey pixels designated as cells, while airspace can be either black or light grey pixels (where brightness of the grey is proportional to concentration of the solution). Thin light grey lines in between cells observed in **g**, **h** and **i** marks cell boundaries. Semi-automatic segmentation was applied to the greyscale images to yield the matching binaries (below greyscale), which attempted to assign white pixels to the cells and black pixels to the background. All scale bars present are set at 300 µm

The working concentration of the cesium iodide solution was determined experimentally by embedding solutions of up to 10% w/v concentration in Kanzi hypanthium samples with a pulsed vacuum profile. 2.75 µm voxel resolution scans were obtained to demonstrate the effect of the contrast agents on the pixel intensity of cell boundaries and contrast filled air spaces (Fig. 1). As the ability to properly define intercellular boundaries relies on the detectable intensity differences between the cell fraction (grey pixels) and the contrast agent (light grey to white pixels). When greyscales were binarized based on pixel intensity with assistance from a Sobel edge detection operator, it became obvious that solution concentrations under 5% resulted in incomplete edge detection (data not shown) than those returned by solutions over 5%. The shortcoming is especially obvious at 1% cesium iodide concentration, where the noise floor is high and the boundaries were nearly undetectable. Therefore, it was unsurprising that the datasets were not useful for further processing. Working concentration of cesium iodide were set at 10% as a result of this experiment set.

### Optimization of contrast delivery protocols

As handling of fragile fruit samples inevitably induces certain degree of damage, testing protocols were selected based upon the fragility of the sample. In order to minimize the risk of sample damage, contrast delivery was first done passively with 10% cesium iodide solution. Only in the event that passive delivery failed to yield sufficient results, was the more harsh active protocol utilized. In any case, both delivery protocols were explored and optimized per sample type as needed (Table 1). Conditions such as incubation time, vacuum pressure, pulse profile was determined via a combination of internal and published work [24, 25]. The chosen protocols were selected based on the ability to detect or mark cell edges in the sample center and high density clusters, as well as the feasibility of image processing protocols from the datasets with experimental workflows in Avizo 9.2.

**Table 1:** Summary of experimental protocols used for contrast delivery

Samples	Incubation timeframe		Quick summary	
	Duration	Successful?^a^	Remarks	Active delivery necessary?
*Passive delivery*				
Apple hypanthium	30, 60, 90 min	–	Inconsistent and incomplete contrast penetration to center of sample, some edges detectable but fragmented	+
Pear hypanthium	30, 60, 90 min	+	Consistent and sufficient contrast throughout sample, with detectable intercellular boundaries	−
Tomato mesocarp	30, 60 min	–	Sample damage and destruction notable, incubation time/interval too long	−
Tomato mesocarp	5, 10, 15 min	+	Consistent and sufficient contrast throughout sample, detectable intercellular boundaries. Sample damage starts to be observable at 15 min	−
Tomato petiolule	30, 60, 90 min	+	Consistent and sufficient contrast throughout sample, detectable cell edges in parenchyma and collenchyma	−
Tomato leaflet	1, 2, 3, 4, 5 h	–	No detectable separation between mesophyll cells	+
Tomato leaflet	10 h	–	Sample oversaturated with contrast solution	+

Samples	Vacuum profile		Results
	Duration + pressure	Successful?	Remarks
*Active delivery*			
Apple hypanthium	[3 min @ 20 kPa, 10 min @ ambient] X2	−	Incomplete penetration to center of samples, edges detection inconsistent in cell clusters
Apple hypanthium	[5 min @ 20 kPa, 15 min @ ambient] X3	+	Consistent contrast movement through sample with easily detectable edges
Leaflet	[3 min @ 20 kPa, 10 min @ ambient] X2	−	Sample destabilized, movement artifacts frequent and severe. Large sections of leaflet flooded with contrast with no detectable intercellular boundaries

^a^Trials were considered successful if the contrast agent were distributed evenly and consistently throughout experimental sample and intercellular boundaries in cell clusters were detectable via a Sobel operator

### Active delivery of contrast and potential detrimental effects of vacuum delivery

**Fig. 2:**
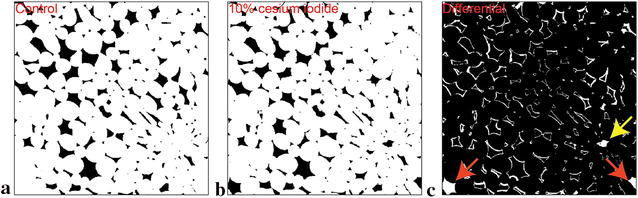
Comparison of segmented binary images from matching control (**a**) and contrast enhanced (**b**) scans of apple (cv. Kanzi) hypanthium tissue. White pixels and black pixels represent cells and intercellular air spaces in (**a**, **b**). **c** Depicts the result of a XOR logical operation, where matching pixels of the two datasets from **a** and **b** are represented by black pixels, while mismatches are represented by white pixels. Red arrows highlights fluid filled intercellular spaces (caused by sample excision and manipulation), as the contrast solution can diffuse into the extracellular fluid (thus differentiating cellular and non-cellular materials). The yellow arrow highlights contrast embedded vasculature (ref to Fig. 3i for 3D representation)

Passive delivery was insufficient for apple hypanthium samples due to incomplete and inconsistent contrast solution migration. Thus, an adapted pulsed vacuum profile was utilized to deliver the contrast solution throughout the sample. Although the modified vacuum protocol was successful, the derived dataset must be assessed for damage or distortion due to the harshness of the protocol itself. To accomplish this, a cylindrical sample of apple hypanthium was scanned prior to and following contrast incubation with the vacuum impregnation protocol. The resulting images stacks were registered and resamples to common spatial coordinates, and a central VOI was cropped out for processing. Binary images of the cells (white) and air spaces (black) from scans pre and post contrast enhancement are shown in Fig. 2a, b. While differences between the two volumes (white, Fig. 2c) exist, the differences between even with the inclusion of vasculature and partially flooded airspaces was 7.8%. However, it was determine that air spaces and vasculature occupies 1.7% of the VOI, as they are now distinguishable from the remainder of the structures. The real difference between cells in terms of volume is approximately 6.1%.

### Passive delivery of contrast via diffusion

Trials with pear hypanthium samples were done in 30 min incubation intervals for the passive protocol. Contrast was insufficient at 30 min of incubation due to the lack of definition towards the sample center. Extending the incubation time to 60 min resulted in sufficient edge detection in the center of the sample, and extension to 90 min yielded little improvement. Similarly, tomato outer mesocarp samples were tested in 30 min initially. However, sample damage and/or destruction was evident at the 30 min mark. Reducing the incubation time to 20 min still yielded significant sample damage. Thus, the intervals were shortened to 5 min and retested up to the 15 min mark. At the 10 min mark, definition was sufficient in the sample center in which edge detection was possible. While extension of the incubation time to 15 min yielded no discernable improvements in boundary definition, while damage within the sample increased notably. Therefore, passive contrast delivery for pear and tomato samples are set at 60 and 10 min respectively.

Unlike pear and tomato samples, leaf and petiolule samples were incubated with the superstructure intact. Natural transpirational pull was exploited to enhance contrast delivery to the non-homogenous samples. Petiolule samples were excised from the leaf in 30 min incubation intervals. Core conductive vessels was visible at 30 min, while intercellular boundaries remained largely unobservable. Extension of the incubation to 60 min resulted satisfactory results, while increase to 90 min resulted in negligible gain in contrast. Leaf blade sections unfortunately yielded unsatisfactory results in all tested intervals and protocols. Initial testing indicated that 30 min intervals was largely insufficient for leaf blade sections, as was the case for 1 h intervals up to 5 h. Even at 5 h, barely detectable changes in the veins were observed in the tomographs, and no discernable increases in contrast was noted. Leaving the sample overnight for 10 h resulted in a highly dehydrated sample with distorted dimension where leaf thickness was less than 50% of the control. Attempts were made with excised leaf blade sections incubated with a vacuum protocol. However, sample stability was insufficient for a high quality scan (internal testing data, not shown). Patches of contrast flooded cells along with movement artifacts provided no notable gain in contrast. Thus concluding the use of the cesium solution to enhance contrast in leaf samples.

### Analysis of contrast enhanced fruit tissue images

**Fig. 3:**
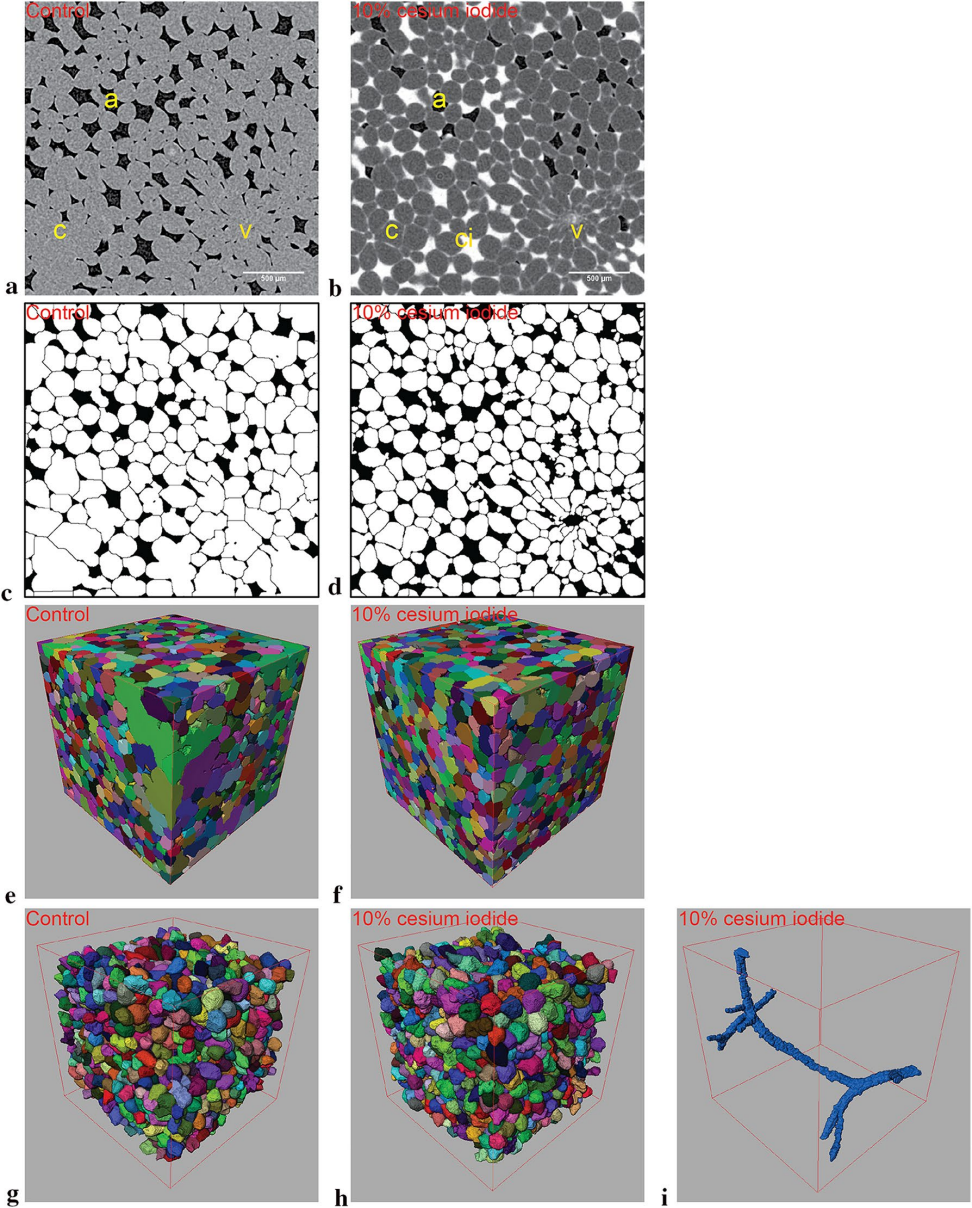
Registered comparison between conventional and contrast enhanced scans of apple (cv. Kanzi) hypanthium (left and central columns respectively). Greyscale images of the conventional scan **a** contains air spaces “a” in black pixels, as well cells “c” and vasculature “v” in grey pixels. Contrast enhanced greyscale image **b** is composed of air space “a”, cells “c”, vasculature “v” as well as contrast solution “ci”, which is represented by black, grey, light grey and white pixels respectively. Note that the contrast solution occupies not only intercellular spaces (marked by light grey lines), but a majority of the intercellular spaces were also invaded by the solution (such as the airspace marked by “ci”). **c**, **d** Depicts segmented cells in white. **e**, **f** Depict the segmented cell volumes in 3D with arbitrary colors, where individual cells in the same neighborhood are marked with a different color. **g**, **h** Remove debris and incomplete cells that intersected the VOI boundaries in the respective control and contrast enhanced datasets. **i** Demonstrates the 3D localization of the vasculature bundle found within the scanned sample. All scale bars present are 500 µm while all bounding edges are 2400 µm

Representative images of apple hypanthium scans prior to and following contrast enhancement are shown in Fig. 3a, b. The registered, binarized, watershed transform separated and labelled cells are shown in 3D (Fig. 3c–h) after removal of incomplete objects in 3D. Moreover, as contrast enhancement allowed vasculature segmentation, 3D plots demonstrate the connectivity and localization of the structure (Fig. 3i). Qualitatively, the dataset demonstrates a notable contrast improvement at cellular boundaries, where segmentation resulted in a notable reduction of large and irregular cell clusters in the corresponding 3D renderings. Quantitatively (Table 2), filtering was applied to labelled objects within the VOI to exclude debris, as well as unreasonably large cells/clusters as well as geometrically unlikely cells (with lower and upper equivalent spherical diameter of 40 and 200 µm, and sphericity index of greater than 0.75 remain). Recovery of lost cell volume was done by label dilation to the reference provided by the Sobel operator to compensate for the imperfect initial segmentation. Even then, the contrast enhanced scans only yielded a 17.5% increase in analyzable volume, along with 0.5% of the sample volume belonging to the vasculature. However, contrast enhancement increased the number of analyzable objects from 951.3 ± 88.9 to 1312.8 ± 140.3, while mean equivalent spherical diameter increased insignificantly from 186.3 ± 5.5 versus 195.8 ± 2.1 µm. Overall improvement due to contrast enhancement in apple hypanthium samples is quantitatively insignificant. Although we were able to increase the analyzable cell volume and cell count, there was an insignificant change in mean cell size.

**Table 2:** Summary of quantitative analysis of VOIs after border kill, filtering by equivalent diameter and sphericity, % volume of vasculature and/or brachysclereids, as well as percent increases in both retained cell volume and count

Samples	Border killed cell volume		Filtered cell volume			Vasculature + brachysclereids volume		Cell count		
	Volume%	SEM	Volume%	SEM	% increase	Volume%	SEM	Number	SEM	% increase
Kanzi (n = 4)										
Control	65.13	1.12	23.23	2.28	–	–	–	951.25	88.95	–
Enhanced	72.57	0.60	26.79	3.05	17.51	0.52	0.17	1312.75	140.35	38.00
Conference (n = 4)										
Control	60.96	0.64	21.88	0.54	–	–	–	1060.25	15.47	–
Enhanced	74.14	0.29	39.46	1.11	85.44*	1.11	0.05	2540.25	12.86	139.59*
Boneparte (n = 4)										
Control	42.06	1.86	35.72	1.74	–	–	–	114.25	18.19	–
Enhanced	49.63	1.93	45.74	3.64	37.98*	3.55	0.84	162.25	23.26	42.01

Statistical significance denoted by * where p < 0.05

**Fig. 4:**
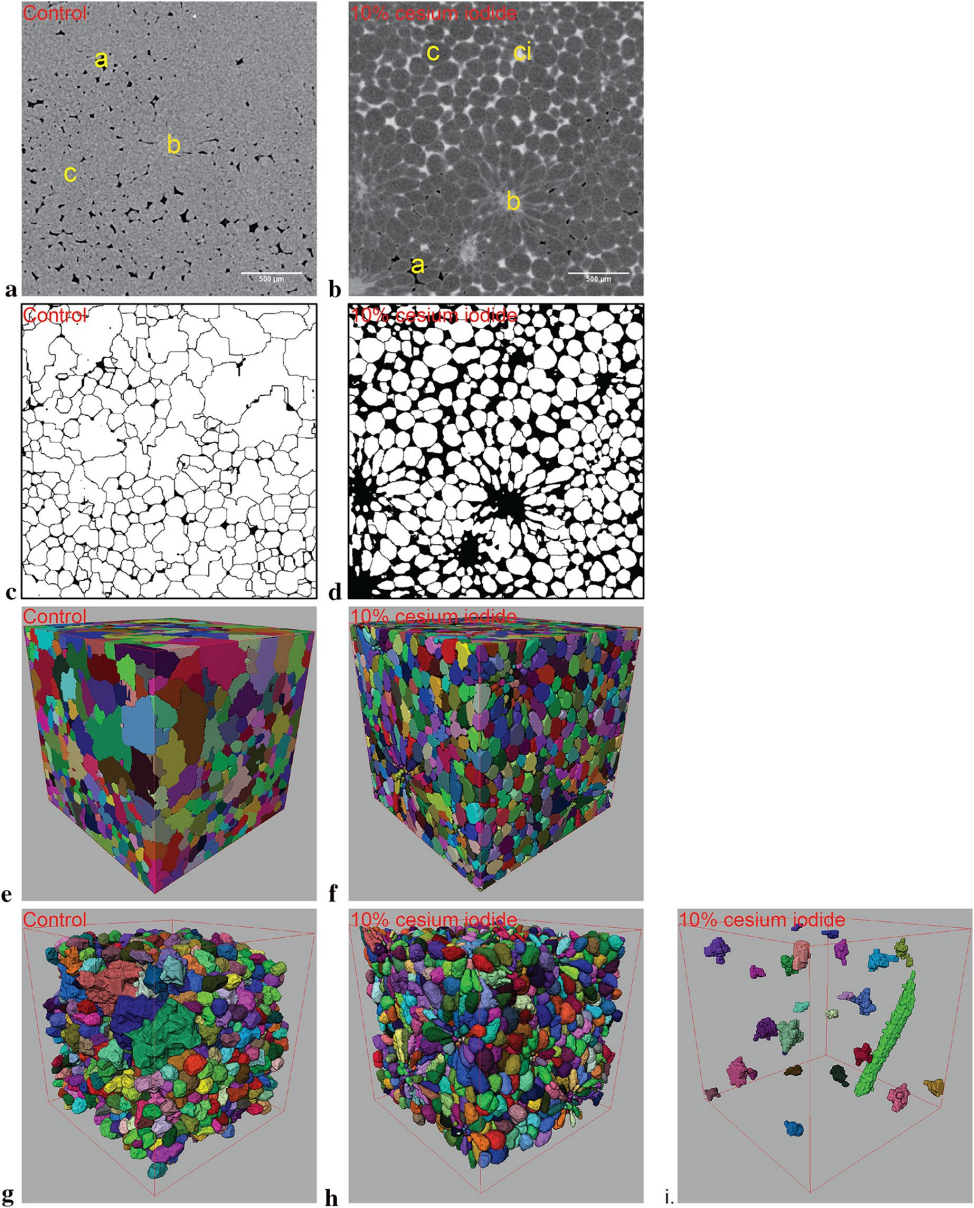
Non-registered comparison between conventional and contrast enhanced scans of pear (cv. Conference) hypanthium (left and central columns respectively). Greyscale image of the conventional scan **a** contain air spaces in black pixels, as well as cells and brachysclereids in grey pixels labelled by “a”, “c” and “b” respectively in the image. Greyscale image of contrast enhanced scan **b** contain black pixels marking air spaces “a”, dark grey pixels marking cells “c”, light grey pixels in clusters marking brachysclereids “b”, near white pixels marking contrast flooded air spaces “ci”, as well as light grey pixels in lines marking intercellular boundaries. **c**, **d** Depict the segmented cells in white. **e**, **f** The segmented cell volumes in 3D with arbitrary colors, where individual cells in the same neighborhood are marked with a different color. **g**, **h** Removes incomplete cells that intersected the VOI boundaries in the respective control and contrast enhanced datasets. **i** The approximate size and localization of the brachysclereids along with a vascular bundle (elongated green structure) in the VOI. All scale bars present are 500 µm while all bounding edges are 2400 µm

Pear hypanthium samples enhanced with cesium iodide yields significant changes in results both qualitatively and quantitatively. However, the delicate nature of pear samples made it infeasible to scan the sample both prior to and following contrast delivery due to handling damage. Regardless, the contrast enhanced tomographs along with the binarized and 3D rendered volume demonstrates a notable improvement in the quality of the datasets compared to the control datasets (Fig. 4). Most notably, the presence of large objects with equivalent spherical diameters of over 500 µm and sharp geometries have diminished (Fig. 4c, e, g). Moreover, brachysclereids (stone cells) with their dense radial cluster of parenchymal cells surrounding them can be segmented with contrast enhancement. The combined average of these stone cells are 1.1% of the overall cell volume, and varies depending on the VOI chosen. Quantitative analysis of pear hypanthium, however, demonstrates the degree of improvement of the contrast enhancement protocol (Table 2). First, the volume of cells with reasonable size and geometries after filtering (equivalent spherical diameter of under 200 µm, sphericity index above 0.75) rose by 85.4% along with a significant increase in cell count to 2540.3 ± 12.9 from 1060.3 ± 15.5 of control samples. Unsurprisingly, 139.6% increase in cell count along with a smaller 85.4% increase the volume of cells resulted in a reduction in cell sizes to 158.2 ± 0.9 versus 192.6 ± 0.2 µm found in the control datasets. Lastly, the average anisotropy of the cells in the enhanced scans is significantly higher compared to that of conventional scans at 0.70 versus 0.64 respectively.

**Fig. 5:**
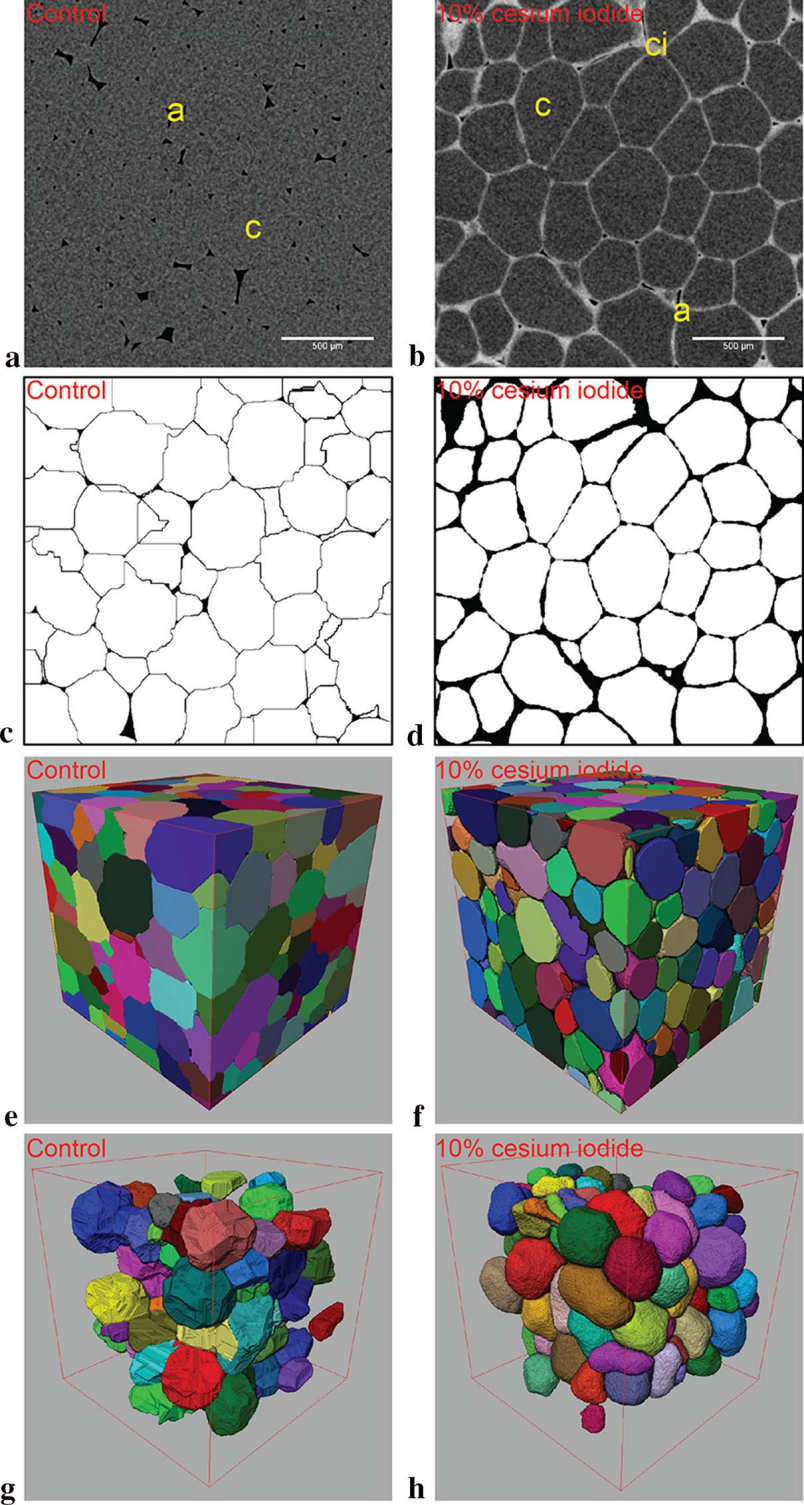
Non-registered comparison between conventional and contrast enhanced scans of tomato (cv. Bonaparte) mesocarp (left and right columns respectively). Greyscale image of the conventional scan **a** contains air “a” labelled by black pixels, while cells “c” are labelled by dark grey pixels. Greyscale images of the contrast enhanced scan **b** contains air “a” labelled by black pixels, cells “c” labelled by dark grey pixels as well as contrast fluid “ci” labelled by near white pixels in intercellular airspaces and boundaries. **c**, **d** The segmented cells in white. **e**, **f** Depicts the segmented cell volumes in 3D with arbitrary colors assigned, where separate cells in the same neighborhood are marked with a different color. **g**, **h** Removes debris and incomplete cells that intersected the VOI boundaries in respective control and contrast enhanced datasets. All scale bars are set at 500 µm while all bounding edges are 2000 µm

Similar to pear hypanthium samples, tomato mesocarp tissue is extremely delicate and thus renders scans prior to and following contrast enhancement infeasible. Regardless, resulting tomographs along with binarized and 3D renderings demonstrates the effect of contrast enhancement (Fig. 5) both qualitatively and quantitatively (Table 2). Control images (Fig. 5a, c) demonstrates the shortcomings of conventional scans where no detectable cell boundaries are present in the tomographs. Furthermore, sharp angles can be observed on the rendered cell surfaces (Fig. 5e, g). Conversely, contrast enhancement yields clear cell boundary information (Fig. 5b, d), as well as significantly smoother cell surfaces in 3D renderings (Fig. 5f, h). The application of a 100 µm debris filter and a sphereicity index cutoff of 0.75, resulted in a significant increase of 38.0% was observed in the volume of reasonably sized and shaped cells within the VOI. However, neither cell count nor cell diameter changed significantly. The cell count increased slightly from 114.3 ± 24.7 to 162.3 ± 23.3, while cell volume decreased from 348.8 ± 19.4 to 331.4 ± 18.8 µm in control and contrast enhanced scans respectively. Thus, contrast enhancement within tomato mesocarp samples is limited to qualitative gains along with notable gains in the volume of cells after filtering.

### Contrast enhancement of petiolule and leaflet sample images

**Fig. 6:**
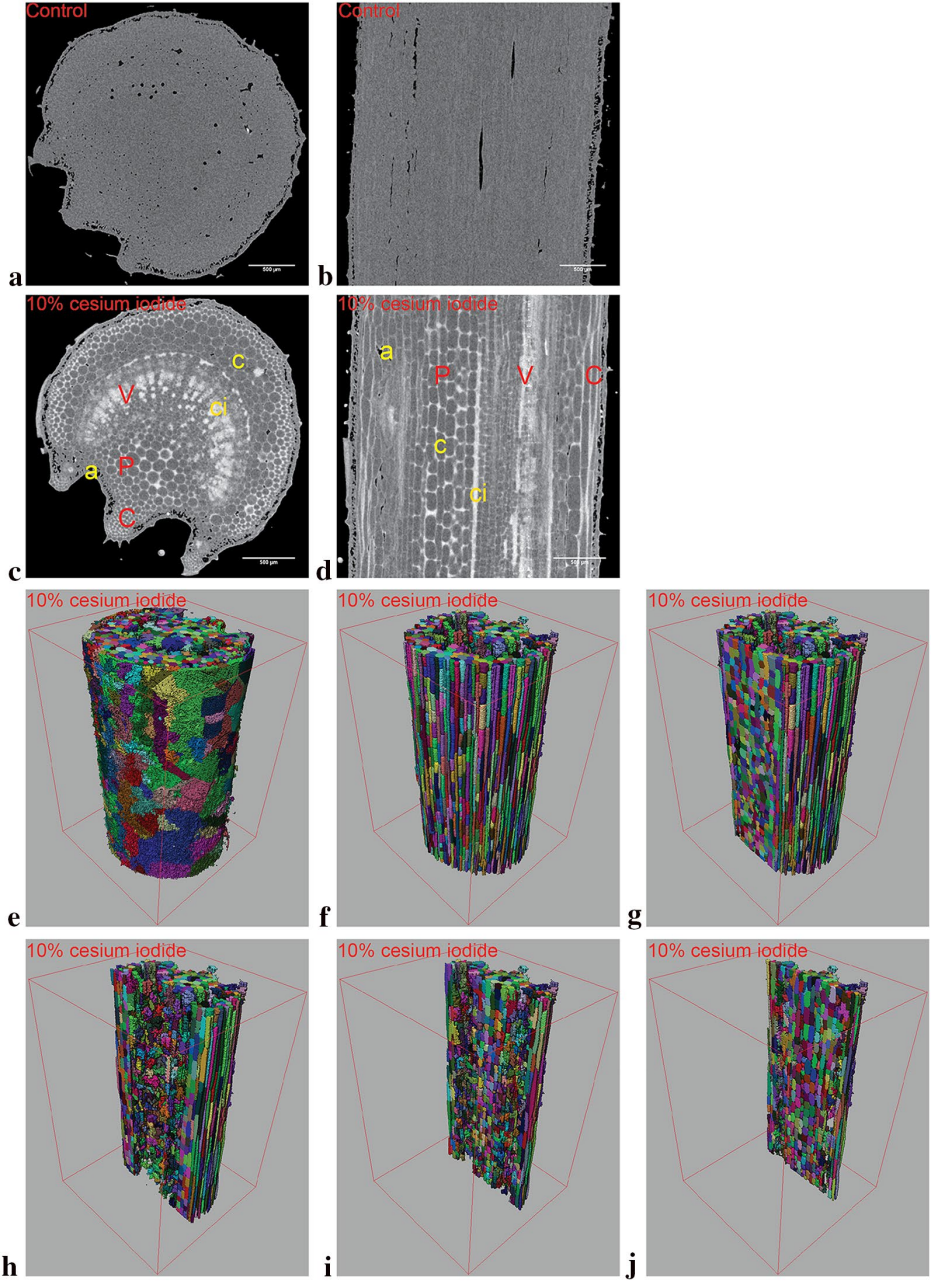
Cross and longitudinal sections of tomato (cv. Merlice) leaf petiolule (**a**, **c** and **b**, **d** respectively) from conventional and contrast enhanced scans (**a**, **b** and **c**, **d** respectively). Sections from conventional scans (**a**, **b**) contain air spaces marked by black pixels, while grey pixels marks plant tissue. Sections from contrast enhanced scans (**c**, **d**) contain air spaces “a” in black pixels, cellular materials “c” in dark grey pixels as well as contrast solution “ci” in light grey pixels. Internal structures such as the vasculature “V”, parenchyma “P” and collenchyma “C” cell layers are distinctively visible. The enhanced greyscale images were segmented (not shown) and rendered in 3D with the vasculature removed but dermal layers left intact (**e**). Removal of the dermal layers resulted in the ability to view the individual cells of the parenchyma and collenchyma (**f**), where separate cells in the same neighborhood are marked with a different color. The rendered volumes were clipped at 750, 1250, 1750 and 2000 µm (**g**–**j**) from the bounding box edge to demonstrate the variance at different sample depths. All scale bars are set at 500 µm while the bounding box is 3000 × 3000 × 4500 µm in size

Standard tomographic scans of leaf petiolule (leaf stems) only yields data on the size and localization of air pockets, epidermal tissue and mineral crystal deposits. Cells of the epidermal layer, collenchyma and parenchyma can neither be quantified nor localized, and no data was available on the location of the vasculature (Fig. 6a, b). Attempts to segment the greyscale images resulted in biologically irrelevant divisions in terms of the number of distinct objects, object size as well as shape. Conversely, contrast enhanced scans yielded cross sectional and longitudinal slices (Fig. 6c, d) with the epidermis, collenchyma, parenchyma and endodermis that are clearly distinguishable from each other. However, the qualitative improvements were still limited to the inner cell clusters. The method did not succeed in providing intercellular separation for the epidermis, nor was there sufficient resolution to properly isolate the vascular bundles (Fig. 6e). Regardless, after discarding unresolved structures, the inner cell clusters of the collenchyma and parenchyma can be segmented with relative ease to yield 3D data. Thus quantitative geometric parameters such as length, width, volume, surface area, sphericity are now available for further analysis in addition to the qualitative improvements.

**Fig. 7:**
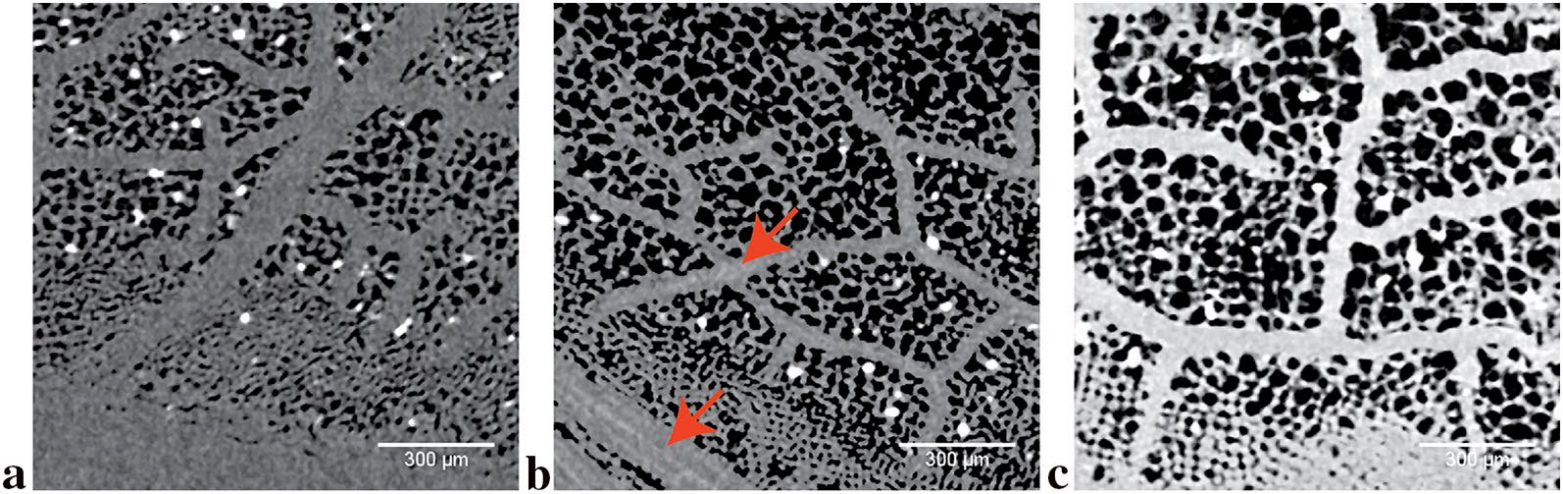
Split section greyscale images of tomato leaflet with no contrast enhancement (**a**), 5 h (**b**) and 10 h (**c**) of contrast incubation by transpirational pull. In the unenhanced greyscale (**a**), leaf tissue is denoted by dark grey pixels, while air and crystalline deposits are marked by black and white pixels respectively. After 5 h of contrast incubation (**b**) faint traces of the contrast solution (light grey lines, see red arrows) are visible in the vasculature of the leaf (marked by grey pixels). After 10 h of incubation (**c**), the contrast solution invaded the plant tissue and oversaturation of the sample occurs. Leaf tissue is now replaced by light grey pixels, and no improvements to intercellular boundaries was observed over the conventional scans. All scale bars are set at 500 µm

Conversely, leaflet datasets experienced neither qualitative nor quantitative improvements even with contrast delivery. This is simply due to the fact that the contrast solution provided virtually no improvements to structural separation in any of the tested leaflet samples. While split sections (parallel to leaf surface) of the leaves show the spatial distribution of the mesophyll cells along with the vasculature (Fig. 7), segmentation of individual cells were largely fruitless. Comparing no contrast with 5 and 10 h contrast embedded samples (Fig. 7a–c respectively), only vasculature can be somewhat apparent after 5 h of contrast delivery (Fig. 7b). Even then, segmentation proved to be immensely difficult even with Sobel edge detection, and was abandoned due to impracticality. Furthermore, the contrast solution diminishes the ability to isolate and analyze mineral crystal deposits overtime. Thus reducing the level of quantitative analysis that can be done on the dataset. Similarly, cross sectional images corroborates such observations (data not shown due to redundancy). Where epidermal cells could not be differentiated from mesophyll cells, and palisade and spongy mesophyll cells cannot be separated from each other.

## Discussion

Porous plant specimens can be easily examined with X-ray micro CT with minimal effort, as there exists a thousand fold difference in the attenuation of air and soft tissue. In the ideal scenario, no sample preparation is necessary and the resulting data is of good accuracy like those previously reported [5]. However, substantial image processing can lose up to 74% of the cell volumes within the VOI depending on the fruit variety of interest [5]. Moreover, given that plant tissue is often not so porous, the power of porosity based image processing rapidly diminishes, and cell clusters have no discernible contrast. Thus, the current study aimed to overcome the limitations frequently faced by micro CT scanners by means of intercellular separation via a cesium iodide solution with minimal sample preparation. Definition improvements to the tomographs would in effect, improve the overall quality of subsequent downstream studies.

### Cesium iodide delivery is relatively simple and flexible

High reproducibility is necessary to ensure the quality of the data generated by new protocols, simpler protocols are thus better to minimize the error rate. As demonstrated by the experimental results thus far, the contrast enhancement protocol itself does not contain inherently difficult steps. Moreover, tissues of varying porosities at 20, 10 and 5%, being Kanzi apples, Conference pears and Bonaparte tomatoes were respectively tested [27–29] alongside tomato leaf petiolule to demonstrate protocol versatility. Even with increasing fragility of the samples going from apples to tomatoes, the cookie cutter approach to contrast enhancement requires only two optimization steps. The first being the duration of the contrast incubation, while the second being the necessity of vacuum impregnation techniques. Furthermore, the resulting detail of larger structures such as vasculature is comparable to previous studies [13], and the degree of detail on a cellular level is markedly better than those at similar resolutions [21]. This is especially notable considering the typical contrast incubation period is under an hour. While the incubation time is quite comparable with lower resolution studies on vasculature [13], it is however in stark contrast to previous high resolution studies where the incubation time was measured in days if not weeks [21, 22]. This combined with the minimal sample preparation further reduces overall experimental time as well as distortions caused by sample preparation steps such dehydration and fixation. Similarly, the short incubation time in conjunction with powerful image processing workflows has the added benefit of preventing significant sample shrinkage and damage found in classical soft tissue contrast studies [30, 31]. That said, the sample preparation and mounting steps used in this study requires a high degree of care by the researcher. Firmly wrapping the samples with parafilm as gently and precisely as possible requires both steady hands and patience. If done correctly, reproducibility is not affected and compressive physical sample distortion can be minimized or eliminated. The subsequent difference in sample volumes should be around (or less than) 6.1% as observed in our study. Which is substantially better than that of the typical iodine based contrast protocols that induces up to 70% sample shrinkage in soft tissue [30]. Nevertheless, the contrast protocols utilized by this study are demonstrated to be simplistic and flexible, and can be used as a rough starting template for sample specific optimizations.

### Image segmentation of cesium iodide enhanced scans is still complex, but more realistic

The ability to segment images based on pixel intensity rapidly diminishes when homogeneity of the material increases. Thus, watershed separation, while powerful [32], loses its effect when moving away from highly porous materials such as apples. Unsurprisingly, cell clusters with poor contrast presents itself as a significant challenge to image segmentation. Where resulting 3D data from standard watershed workflows contain angular outer geometries, unrealistic sizes and clustering. Thus, manual segmentation must be used if possible, at a cost of increased time consumption and lower replicability.

The cesium iodide overcomes the limitation faced by standard image processing protocols by increasing the pixel intensity of intercellular spaces, thus effectively marking cell boundaries. Ideally, the enhanced contrast would allow simple intensity based segmentation. However, the size of intercellular boundaries being much less than 5 microns (which is effectively up to two pixels wide in this study) places severe constraints on such simplistic workflows. Although image processing workflows cannot be simplified from those previously used [5], the accuracy of the datasets is notably improved. As an example, consider the pear hypanthium data presented. Instead of having a low volume of unrealistic cells along with large aggregate clusters, contrast enhanced image processing yields data on actual cellular boundaries. Quantity and localization of stone cells and vasculature is an added bonus from the workflow and adds to the value of such processing methods. Additionally, components such as cells, brachysclereids and vasculature all have distinctive characteristics with varying anisotropy, length, diameter, surface area and volume. Combination of these parameters can be utilized to extract interesting 3D data such as size and directionality of parenchymal cells surrounding brachysclereids, the brachysclereids themselves as well as vascular connectivity. Obviously, this is impossible to obtain without the contrast enhancement regardless of image processing workflow. All of which is a testament to the value of the enhancement properties of the contrast protocol.

However, it must be mentioned that care must be taken when processing enhanced datasets, as cell volume recovery is sometimes necessary since the contrast solution has a tendency to mark cell boundaries slightly thicker. Regardless, the segmentation and image processing workflow have been internally validated against a manual segmentation workflow as described in a previous publication [5]. Both automated and manually segmented data did not differ significantly (internal testing, data not shown), which is unsurprising since the process is only semi-automatic and manual input is required to ensure data integrity.

### Cesium iodide contrast enhancement is more successful with denser or heterogeneous tissue types

As previously eluded to, segmentation difficulty of reconstructed images is roughly inversely proportional to the porosity of the sample. The porosity of apples can be greater than 20% [28], thus watershed assisted segmentation is reasonably accurate. Unsurprisingly, the degree of improvement provided by the contrast agent is only a 17.5% increase in cell volume that can be analyzed. Moreover, the minor changes to cell count and diameter puts them in line with previous reported figures [5]. While contrast enhancement is limited to qualitative gains for highly aerated samples, vascular mapping is now possible with the new protocols.

Conversely, tomatoes and pears have respective porosities of under 5 and 10% [27, 29]. This is complicated by the large cell count and cell volume of pear and tomato samples [5, 33], which challenges the feasibility and accuracy of existing segmentation techniques. Thus, the reliance on resource heavy methods such as microscopy [34, 35] and synchrotron based scanners [5, 27] is perfectly understandable. This study demonstrates that these expenses can be largely circumvented by applying the contrast protocol. In pear samples, both cell count and total cell volume that can be segmented increases significantly from contrast enhancement. Moreover, stone cells, surrounding parenchyma cells, and vasculature can be resolved from the reconstructed images, thus providing valuable information that is previously not obtainable. This makes the enhancement protocol highly worthwhile for use with similar samples as both qualitative and quantitative gains are obvious. For tomato samples, the gains were more qualitative than quantitative. Consider Fig. 5g, where cell boundaries are highly angular which deviates from previous 2D and 3D imaging work [36, 37]. The feasibility of utilizing these segmented images for downstream studies is limited. Conversely, contrast enhancement yields more plausible surface geometries along with an increase retained cell volume after filtering. This significantly increases the plausibility of utilizing such datasets for downstream studies. Unsurprisingly, highly variable cell sizes in the outer mesocarp [36] limited the quantitative significance of the increases in cell count. Nevertheless, contrast enhanced scans can provide more reasonable 3D data in terms of cell size, shape, orientation when compared to microscopy data. Even advanced methods such optical granulometry [37, 38] has severe limitations, and is bound by microtome section thickness, sample preparation time, and destruction of 3D characteristics. These limitations are particularly problematic as mesocarp cells often exceed thickness of microtome slices.

Similarly, leaflet petiolule and conductive macrostructures are low in porosity fractions [39, 40], as their primary function is structural support and fluid transport. The lack of intercellular air spaces meant that segmented images (data not shown) yielded data that are completely unusable for any downstream studies. Contrast enhancement yields 3D data on size, shape, orientation, quantity of collenchyma and parenchyma cells in the petiolule (data not shown) along with vasculature localization. Admittedly, the data on the dermal layers are not useful (Fig. 6e), and vasculature information is minimal due to limited definition. Sufficient quantitative data on the parenchyma and collenchyma were obtained to isolate them. The resulting 3D renderings demonstrates the quantity, orientation and cell diameters with comparable detail compared to similar microscopy studies [41, 42].

### Cesium iodide contrast is subject to limitations

Although contrast enhancement is obvious for most samples examined in this study, the agent itself is not applicable for all sample types.

First, cellular separation by contrast enhancement of highly two dimensional samples such as leaves [43] was not achieved. Thus contrast enhanced scans yielded no discernable improvements over conventional micro CT scans [44]. This is unsurprisingly as the passive relies on transpiration pull to draw the solution across the veins, which is particularly problematic as symplastic pathways exist between vasculature and mesophyll cells [45]. As such, the contrast solution traverses through the cell rather than around them in an apoplastic manner. Extending the incubation time merely allows more time for the solution to diffuse through the cells, rather than bypassing them. Although vacuum impregnation of leaflets were attempted (data not shown), a different set of problems were encountered. The contrast agent is in essence a 10% metallic salt solution, which inherently has the ability to dehydrate, distort and damage the sample. This is typically not an issue with 3D samples, as the outer layer effectively acts as a buffer zone for damage. Leaves however, being two dimensional, did not have this buffer zone and the detrimental effects were almost immediate and sample stability reduced to the point where movement artifacts were rampant. The lack of a sacrificial layer also resulted in unmitigated contrast solution migration, resulting in large patches of contrast flooded mesophyll cells with no contrast improvement. Therefore it is recommended that conventional optical clearing techniques with fluorescent microscopy should be utilized for highly planar samples such as leaves [46].

Second, timing is critical to the success of this contrast enhancement protocol. As the solution is dehydrating and potentially damaging to the sample, an upper limit is inherently present for the duration of incubation. Although the timeframe is typically well beyond what is required for successful imaging. Additionally, protocol timing is reliant on the diffusivity characteristics of the sample. Indeed, Fick’s law stipulates the rate of diffusion is dependent on the path length as well as diffusivity of the encountered path. Large tomato cells grant a shorter path to the center, while small pear cells inevitably prolongs the distance to the specimen center. Furthermore, different samples likely differ in diffusivity in the apoplastic path to the center, which is dependent on composition and physical characteristics of the cell boundaries. In any case, detailed characterization of cell wall composition is well beyond the scope of this study. Thus it is much quicker for the experimenter to run a time series trial to experimentally determine the necessary incubation period for achieving necessary contrast enhancement.

## Concluding remarks

In this paper, cesium iodide is presented as a viable contrast enhancing agent for improving the accuracy of segmented data derived from X-ray micro-CT scans. The benefits of utilizing such a solution are demonstrated in this paper. Although the methodology is currently imperfect, sample type specific optimizations and validation along with more advanced segmentation algorithms in the future can be anticipated to minimize the shortcomings of this contrast solution. This method should prove valuable to the plant science field.
